# Characterisation of HOIP RBR E3 ligase conformational dynamics using integrative modelling

**DOI:** 10.1038/s41598-022-18890-6

**Published:** 2022-09-08

**Authors:** Marius Kausas, Diego Esposito, Katrin Rittinger, Franca Fraternali

**Affiliations:** 1 New Hunt’s House, King’s College London, Guy’s Campus, London, SE1 1UL UK; 2 Molecular Structure of Cell Signalling Laboratory, The Francis Crick Institute, 1 Midland Road, London, NW1 1AT UK; 3 The Francis Crick Institute, 1 Midland Road, London, NW1 1AT UK; 4 Present Address: Illumina Centre, 19 Granta Park, Great Abington, Cambridge, CB21 6DF Cambridgeshire UK

**Keywords:** Biochemistry, Biophysics, Computational biology and bioinformatics, Structural biology

## Abstract

Multidomain proteins composed of individual domains connected by flexible linkers pose a challenge for structural studies due to their intrinsic conformational dynamics. Integrated modelling approaches provide a means to characterise protein flexibility by combining experimental measurements with molecular simulations. In this study, we characterise the conformational dynamics of the catalytic RBR domain of the E3 ubiquitin ligase HOIP, which regulates immune and inflammatory signalling pathways. Specifically, we combine small angle X-ray scattering experiments and molecular dynamics simulations to generate weighted conformational ensembles of the HOIP RBR domain using two different approaches based on maximum parsimony and maximum entropy principles. Both methods provide optimised ensembles that are instrumental in rationalising observed differences between SAXS-based solution studies and available crystal structures and highlight the importance of interdomain linker flexibility.

## Introduction

Multidomain proteins are comprised of two or more domains that fold and function independently. These proteins are highly prevalent in eukaryotic proteomes and regulate numerous cellular processes, including signal transduction, gene expression or protein folding^1,2^. Their modular architecture is often maintained by interdomain linkers that provide the required flexibility for domains to rearrange themselves relative to each other⁠^3–5^. The ability to adopt a variety of conformational states underlies the versatile functional repertoire of multidomain proteins. A key cellular process involving multidomain proteins is protein ubiquitination, a post-translational modification that regulates many aspects of cellular behaviour^6^. Protein ubiquitination is mediated by a cascade of three enzymes, E1 activating, E2 conjugating and E3 ligating enzymes that function in a sequential manner. E3 ligases confer substrate specificity and catalyse attachment of a ubiquitin (Ub) molecule to a lysine residue in substrate proteins often followed by ubiquitin chain extension^7^. The work presented here focuses on the E3 ligase HOIP, which belongs to the RING-in-Between-RING (RBR) subclass of E3 ubiquitin ligases that function via a covalent E3 ~ Ub intermediate. HOIP, together with its accessory binding partners HOIL and SHARPIN, forms the linear ubiquitin chain assembly complex (LUBAC) which synthesizes linear polyubiquitin chains that regulate immune and inflammatory signalling pathway, as well as apoptosis^8–10^. RBR E3 ligases contain a conserved catalytic RBR domain that consists of three sequential zinc-binding subdomains, RING1 (E2 interacting site), IBR (in-between RING) and RING2 (containing the catalytic cysteine active site). The subdomains are separated by two flexible linkers: linker 1 (L1) bridging RING1 and IBR domains and linker 2 (L2) connecting IBR and RING2 subdomains^11,12^.

Substrate ubiquitination by RBR E3 ligases is a dynamic multistep process that often requires release of an autoinhibitory conformation to establish the active enzyme. Catalytic activity is initiated upon binding of the E2 ~ Ub conjugate to RING1, followed by ubiquitin transfer onto the active site cysteine in RING2 to form a thioester intermediate, and final ubiquitin transfer onto a target substrate^12^. The presence of flexible interdomain linkers allows RBR subdomains to adopt multiple conformations relative to each other and is crucial for orchestrating ubiquitin transfer from E2 to substrate. These linkers and their flexibility are central to the function of RBR E3 ligases: (i) in crystal structures, linkers often have higher temperature factors or are disordered, suggesting conformational flexibility^13–17^; (ii) linkers allow relative rearrangement of RBR subdomains as indicated by comparison of structures of autoinhibited and active RBR conformations, where the overall fold of RBR subdomains is conserved, but their relative orientations are distinct^13,18–22^ (iii) solution experiments indicated that RBR/E2∼Ub complexes existed in a conformational equilibrium^23^⁠; (iv) linkers are found to act as protein–protein interaction sites for binding ubiquitin molecules^18,19,24^.

Despite HOIP being one of the most extensively studied RBR proteins, a detailed description of the conformational dynamics of its catalytic RBR domain in solution is currently missing. First structural insight into the dynamic nature of the HOIP RBR domain was provided by the crystal structure of a HOIP RBR/E2 ~ Ub (UbcH5B ~ Ub) complex. In this complex, the HOIP RBR domain adopts two distinct conformational states, an “extended” and a “closed” conformation (5EDV)^18^⁠. The extended conformer is formed by a single polypeptide chain, whilst the closed conformer is formed by two polypeptide fragments that interact with the same E2 ~ Ub conjugate, and which represents the ubiquitin transfer complex (described in more detail below). Based on this structural arrangement we hypothesized that the two conformational states of the RBR domain observed in this crystal structure represent the apo inactive and E2 ~ Ub bound active conformations^18^.

A previous SEC-SAXS-based analysis indicated that the isolated HOIP RBR domain populates mainly extended conformations in solution^23^⁠. Surprisingly, this study further suggested that in solution even in the presence of the E2 ~ Ub conjugate the HOIP RBR retains its high flexibility and preference for extended conformations⁠. Based on these apparently contradictory observations we wondered whether the closed E3/E2 ~ Ub complex observed in the crystal structure may be short-lived in solution and the HOIP RBR domain preferentially populates extended conformational states. Due to flexibility of interdomain linkers, structural characterisation of multidomain proteins can be challenging. To overcome such challenges, a number of integrated structural biology methods were developed^25–28^⁠. Integrative modelling methods provide a way to combine experimental methods and computer simulations to provide atomistic insight into global and local flexibility properties of a biomolecule and produce an ensemble of conformations that is consistent with the experimental data.

In this study, we combined small angle X-ray scattering (SAXS) experiments and molecular dynamics (MD) simulations to investigate preferred conformational states of the HOIP RBR domain in isolation. We applied maximum parsimony (MaxPars; MP) reweighing, to generate a minimal structural ensemble of the RBR domain in accordance with experimental data. Furthermore, we compared MaxPars ensemble with maximum entropy (MaxEnt; ME) reweighted ensemble and found a qualitative agreement between the two approaches, with the MaxEnt ensemble producing a better fit to the experimental data. The ensembles identified a conformational preference of the isolated HOIP RBR domain towards more compact states though without a single preferential arrangement of the domains with respect to one another. This study provides further insights into the dynamic nature of RBR domains in solution and sheds light on the importance of the linkers in this conformational behaviour. Furthermore, it highlights the power of integrative modelling in characterising multi-domain protein conformational dynamics and interpreting experimental data.

## Results

### Small angle X-ray scattering analysis of the HOIP RBR domain

**Figure 1:**
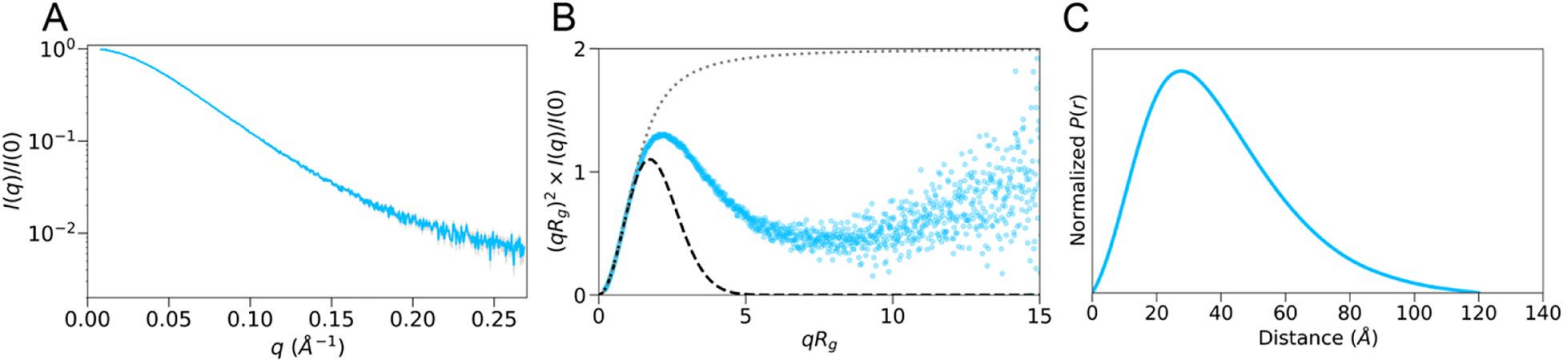
Small angle X-ray scattering analysis of the HOIP RBR domain. (**A**) Scattering plot of observed intensities *I(q)* versus scattering angles *q*. (**B**) Dimensionless (normalized) Kratky transformation of the scattering intensities. For reference, a Guinier approximation of globular molecules at low *q* angles is present as a grey dashed line and plotted as *f(x)* = *x*^*2*^* ∗ exp(− x*^*2*^*/3)*, where *x* is *qR*_*g*_. The random coil is approximated with *f(x)* = *2/x*^*2*^* [exp(− x*^*2*^*)* + *x*^*2*^* − 1]* (grey dotted line), where *x* is *qR*_g_. (**C**) Min–Max normalized *p(r)* pair distribution function.

To investigate conformational dynamics of the HOIP RBR domain in solution, size-exclusion chromatography (SEC) coupled with SAXS (SEC-SAXS) was used (Fig. 1A). We previously performed similar experiments⁠ but collected additional SEC-SAXS data for this study using a newer and more sensitive detector (EIGER X 4 M, Dectris Ltd, Supplementary Table 1). Analysis of the SEC-SAXS data provided a good agreement with previous findings and identified similar structural properties of the HOIP RBR domain in solution^23^ (Supplementary Table 2). From the Guinier approximation, the radius of gyration *R*_*g*_ was 30.3 ± 0.1 Å. Construct flexibility was assessed using dimensionless Kratky analysis^29^⁠. At low *qR*_*g*_, the HOIP RBR domain conferred a broad bell-shaped Kratky profile with a maximum peak close to the peak of Guinier approximation for globular molecules (Fig. 1B). However, the Kratky profile peak was shifted to the right and the upturn of the Kratky profile at higher *qR*_*g*_ values indicates the presence of flexibility (Fig. 1B). The maximal dimension (*D*_*max*_) of the RBR domain was 120 Å, which corresponds to the longest dimension of the extended HOIP RBR conformation observed in the crystal structure (119 Å). Furthermore, the tail of *P*(*r*) pair distribution suggested the presence of extended conformations with long intramolecular distances (Fig. 1C). Taken together, the SEC-SAXS data indicate that the HOIP RBR domain is flexible in solution and occupies extended conformational states in agreement with previous studies.

### Structural models of the HOIP RBR domain

**Figure 2:**
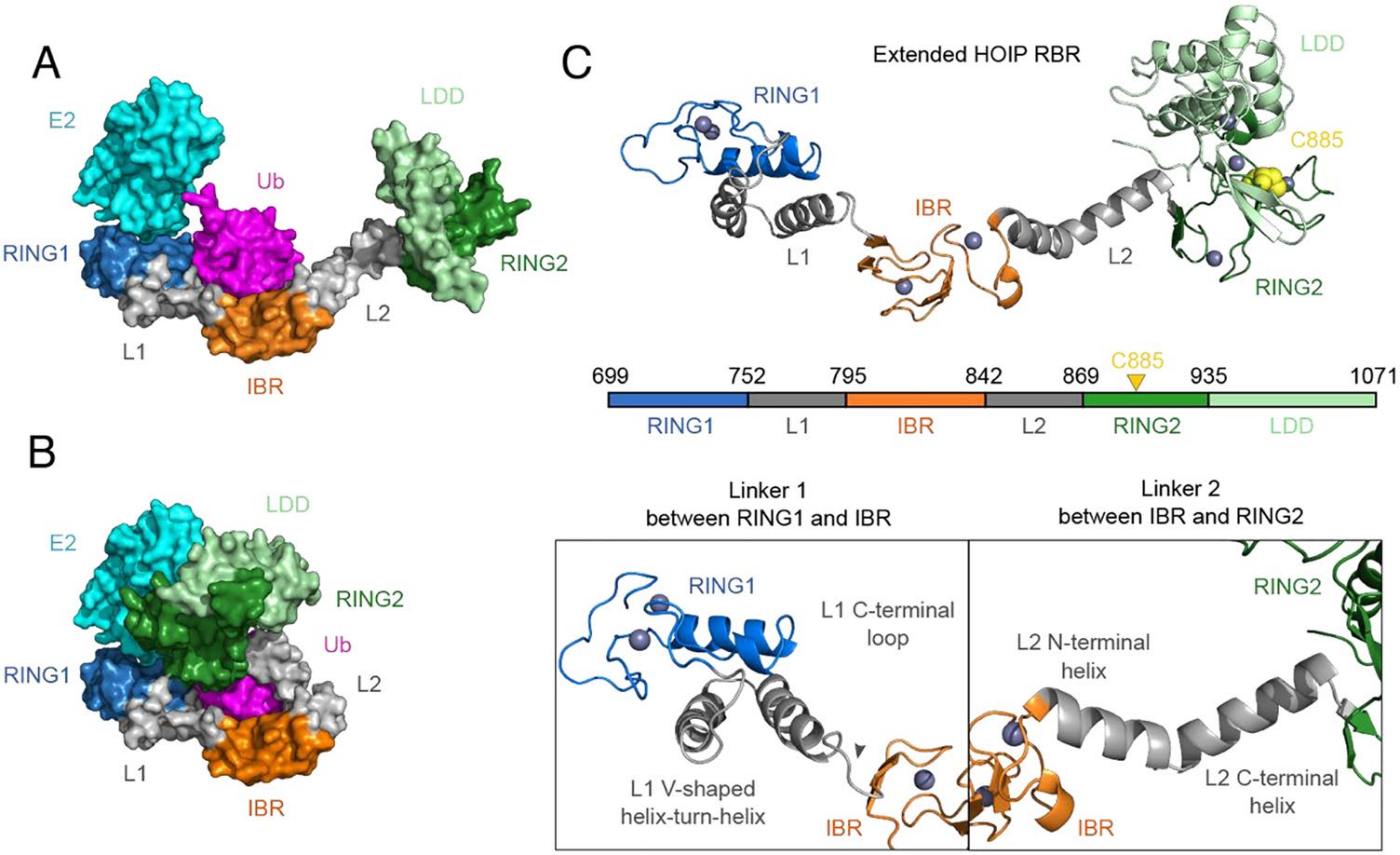
Structural models of the HOIP RBR domain. (**A**) The *extended* and and (**B**) *compact* conformation of HOIP RBR domain observed in the crystal structure (5EDV), respectively. UbcH5B is coloured in cyan and conjugated ubiquitin is coloured in magenta. (**C**) The model of the extended HOIP RBR domain consists of RING1 (699–751), L1 (752–795), IBR (796–841), L2 (842–868), RING2 (869–935) and LDD (936–1071) structural regions. In this study, the RING2-LDD supradomain is referred to as the RING2 domain. L1 adopts a V-shaped helix-turn-helix motif that makes extensive contacts with RING1, whilst the short L1 C-terminal loop connects the helix-turn-helix to the IBR domain. L2 forms two helices, N-terminal and C-terminal, with a bend in the middle. RING1, IBR, RING2 and linkers are coloured blue, orange, green and grey, respectively. The catalytic cysteine C885 is shown as yellow spheres. Zn^+2^ ions are shown as dark blue spheres.

To perform MD simulations of the HOIP RBR domain, we used the extended single polypeptide molecule present in the crystal structure of the HOIP RBR domain in complex with the UbcH5B∼Ub conjugate as a starting model (5EDV)^18^ (Fig. 2A). The asymmetric unit of this E2 ~ Ub/E3 complex structure contains two HOIP RBR molecules that form a dimer in a cross fashion. In this cross-dimer arrangement, each RBR molecule interacts with two UbcH5B∼Ub conjugates at opposite ends (Supplementary Fig. 1A), while each UbcH5B∼Ub conjugate contacts two different regions of HOIP: (i) the RING1-IBR fragment from one HOIP molecule (fragment I, yellow dashed lines) and (ii) the RING2 domain from the second HOIP molecule (Supplementary Fig. 1B, fragment II, red dashed lines). Despite the apparent dimeric nature of the HOIP RBR domain in the asymmetric unit, solution experiments and mutagenesis studies indicate that it is monomeric in solution and dimerization is a crystallisation artefact^18^⁠. The authors of this study suggest that the active E3/E2 ~ ubiquitin transfer complex, captured in this structure is formed by the RING1-IBR fragment of one chain and RING2 of the other that together wrap around the E2 ~ Ub conjugate in a clamp-like manner (Fig. 2B; Supplementary Fig. 1C). Based on this study we hypothesised that this crystal structure can be interpreted as having captured two different conformational states of the RBR domain, which we refer to as *extended* and *closed* conformations. While the extended conformation is represented by a single polypeptide chain (Fig. 2A; Supplementary Fig. 1D in orange), the closed conformation is a composite of two polypeptide chains (Fig. 2B; Supplementary Fig. 1C in orange).

The final starting model of the extended conformation spans residues 699–1071, comprising RING1 (699–751), L1 (752–795), IBR (796–841), L2 (842–868) and RING2 (869–1071) (Fig. 2C). The RING2 domain (869–934) forms a supradomain together with the C-terminal linear ubiquitin chain determining domain (LDD) (935–1071), which is specific to HOIP. For the purpose of the work presented here, we will refer to the RING2-LDD (869–1071) supradomain as the RING2 domain of HOIP, unless otherwise stated.

### Molecular dynamics analysis of HOIP RBR domain

**Figure 3:**
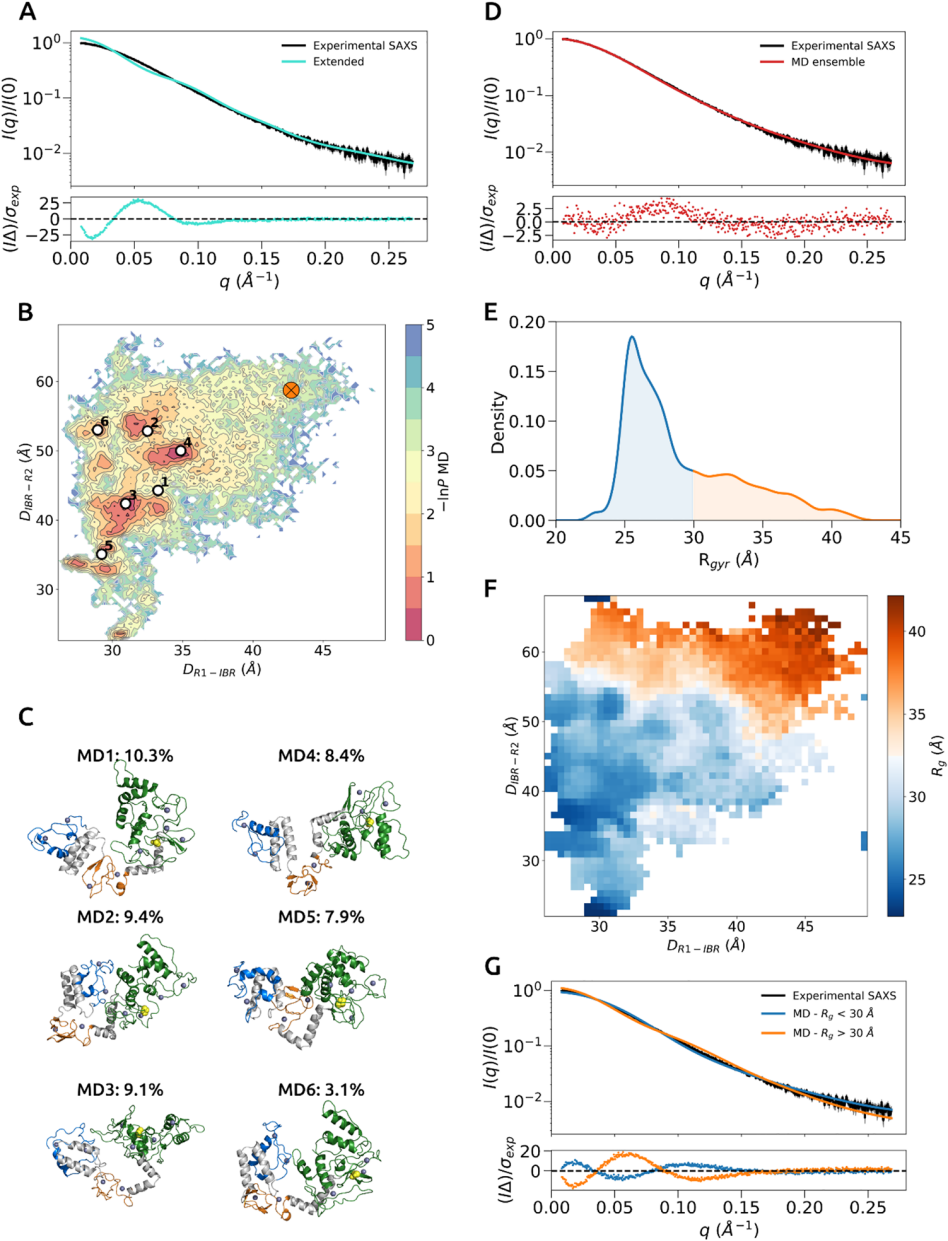
MD analysis of the HOIP RBR domain. (**A**) Agreement between experimental and extended HOIP RBR domain model scattering profiles, which are coloured black and cyan, respectively. The error-weighted residuals (I∆)/σ_exp_ = [I_exp_ (q) – I_ensemble_ (q)]/σ_exp_ versus q are plotted below. (**B**) A free energy surface projected onto two collective variables, D_RING1−IBR_ and D_IBR−RING2_. Starting MD conformation of the extended HOIP RBR domain for molecular simulations is represented as orange circle cross. Representative conformational clusters are marked as white points. (**C**) Selected conformational conformers (MD1-6), where RING1, IBR and RING2 domains are coloured in blue, orange and green, respectively. The catalytic cysteine C885 is shown as yellow spheres. Zn^+2^ ions are shown as dark blue spheres. For an overlap of members in two different orientations see Supplementary Fig. 3A. (**D**) Agreement between experimental and MD ensemble scattering profiles, which are coloured black and red, respectively. (**E**) MD ensemble *R*_*g*_ distribution, where values > 30 Å and < 30 Å are coloured in blue and orange, respectively. (**F**) A free energy surface projected onto two collective variables, D_RING1−IBR_ and D_IBR−RING2_, but coloured according to *R*_*g*_ values. (**G**) Agreement between experimental, MD ensemble frames that have *R*_*g*_ values of > 30 Å and < 30 Å scattering profiles, which are coloured black, blue and orange, respectively. Experimental error is denoted in grey.

We first investigated whether the extended conformation of the HOIP RBR domain as observed in the crystal structure would fit the experimental SAXS data. The calculated theoretical scattering profile of this conformer showed a poor fit to the experimental data with a *χ*^2^_*red*_ value of ~ 121 (Fig. 3A). This suggests that the extended conformation is a poor model to fit the experimental data and it is likely that the RBR domain experiences a dynamic conformational equilibrium. Thus, to investigate the conformational landscape, we used a set of unbiased MD simulations using GROMOS 54A8 forcefield^30–32^ to sample conformational states of the HOIP RBR domain in solution. We performed a total of 10 simulations, each 100 ns in length (1 µs of total simulation time). The conformational sampling was analysed through a set of collective variables, including distances between the center-of-mass (COM) of three domains, *D*_*RING*1−*IBR*_ and *D*_*IBR*−*RING*2_ and radius of gyration *R*_*g*_. The COMs for RING, IBR and RING2 domains were defined based on the 699–751, 796–841 and 869–934 regions, respectively. The initial inter-domain distances (*D*_*RING*1−*IBR*_ and *D*_*IBR*−*RING*2_) and *R*_*g*_ for the starting extended model (derived from the crystal structure) are 42.8 Å, 58.7 Å and 39.6 Å, respectively (*D*_*RING*1−*IBR*_, *D*_*IBR*−*RING*2_, and *R*_*g*_ values for the closed HOIP RBR domain are 42.8 Å, 36.4 Å and 27.1 Å, respectively). The inter-domain distances of the MD ensemble were projected onto a two dimensional free-energy surface (Fig. 3B) and a set of representative conformational clusters were mapped onto observed energetic minima (Fig. 3B,C). The generated conformational ensemble from unbiased simulations indicated predominantly compact conformations of the isolated HOIP RBR domain described by shorter inter-domain distances between subdomains (Fig. 3B). However, there was no preferred conformation within the observed compact conformers of HOIP RBR domain as the relative orientations of RBR subdomains were notably varied with respect to one another (Fig. 3C).

Next, we compared the average radius of gyration R_g_ from the MD trajectories with the experimental measurement. As the SEC-SAXS analysis provides two different R_g_ measurements, Guinier-based and *P(r)*-based values, we chose *P(r)*-based as a more representative value of the radius of gyration of a molecule in solution, because the pair distribution *P(r)* function takes into account all of the SAXS curve and is not limited to the sole Guinier region. The average R_g_ from the MD ensemble was 29.1 +/− 1.1 Å, which was only slightly lower than the *P(r)*-derived value (31.2 +/− 0.1 Å), indicating the good fit of the MD ensemble. To compare the agreement between simulations and experiments, we computed a single scattering profile by averaging over all MD trajectory frames (50,001) and examined the fit to the experimental SAXS data. The MD ensemble had a relatively good fit, with a *χ*^2^_*red*_ value of 2.3 (Fig. 3D). This observation suggests that the previous interpretation of the SAXS data that the RBR domain is dynamic but populates mainly elongated conformations is too simplified and that instead a significant proportion of more compact conformations contributes to the observed scattering profile. To interrogate and validate the simulations further we compared radii of gyration, root-mean-squared deviations (calculated on backbone atoms) and energy landscape representations projected onto the collective variables D_RING1-IBR_ and D_IBR-RING2_ of the extended HOIP RBR domain for each of the 10 × 100 ns simulations (Supplementary Fig. 2). These time-course plots show overall a consistency, for all simulated trajectories, in converging towards a more compact state with lower R_g_ values after equilibration.

To investigate which set of conformers contribute mostly to the fit, we decided to split sampled conformers into two groups displaying R_g_ values below or above 30 Å, respectively. The group with R_g_ values below 30 Å represented largely structures with shorter interdomain distances (Fig. 3F) and made up the largest proportion of R_g_ distribution (Fig. 3E), whilst the group with R_g_ values above 30 Å represented conformers with longer interdomain distances (Fig. 3F) that were in the shoulder of the R_g_ distribution (Fig. 3E). As before, we computed an averaged theoretical scattering profile for each group and compared it with the experimental data. Either group alone (below and above 30 Å) showed poor fits with *χ*^2^_*red*_ values of 14.7 and 51.9, respectively (Fig. 3G). Therefore, a combination of both elongated and more compact states was required to provide a better fit to the experimental data and constitute a more accurate representation of the conformational landscape of the HOIP RBR domain in solution.

### MaxPars ensemble modelling of the HOIP RBR domain

To select an ensemble of conformers from the MD states that accurately reproduced the observed scattering data we applied integrative modelling techniques. We chose to construct a reweighed ensemble using Basis-Set supported SAXS (BSS-SAXS) that employs a MaxPars reweighing framework^33–35^. BSS-SAXS attempts to construct a minimal ensemble with a small number of conformers that represent major conformational states in solution that can explain the experimental data. In the construction of a minimal ensemble, BSS-SAXS employs a two-step clustering approach to select a set of diverse states. The first step consists of a structural clustering to extract a set of representative conformers from the MD simulations. In the second step, a theoretical scattering profile is computed for each representative conformer and resulting profiles are clustered to extract a set of representative scattering profiles, which are termed basis-set. Then, relative weights are assigned to each basis-set member and a weighted average scattering profile is computed to fit the experimental data. Finally, the fit is optimised through sampling of the posterior distribution of weights using a Bayesian Monte Carlo approach.

**Figure 4:**
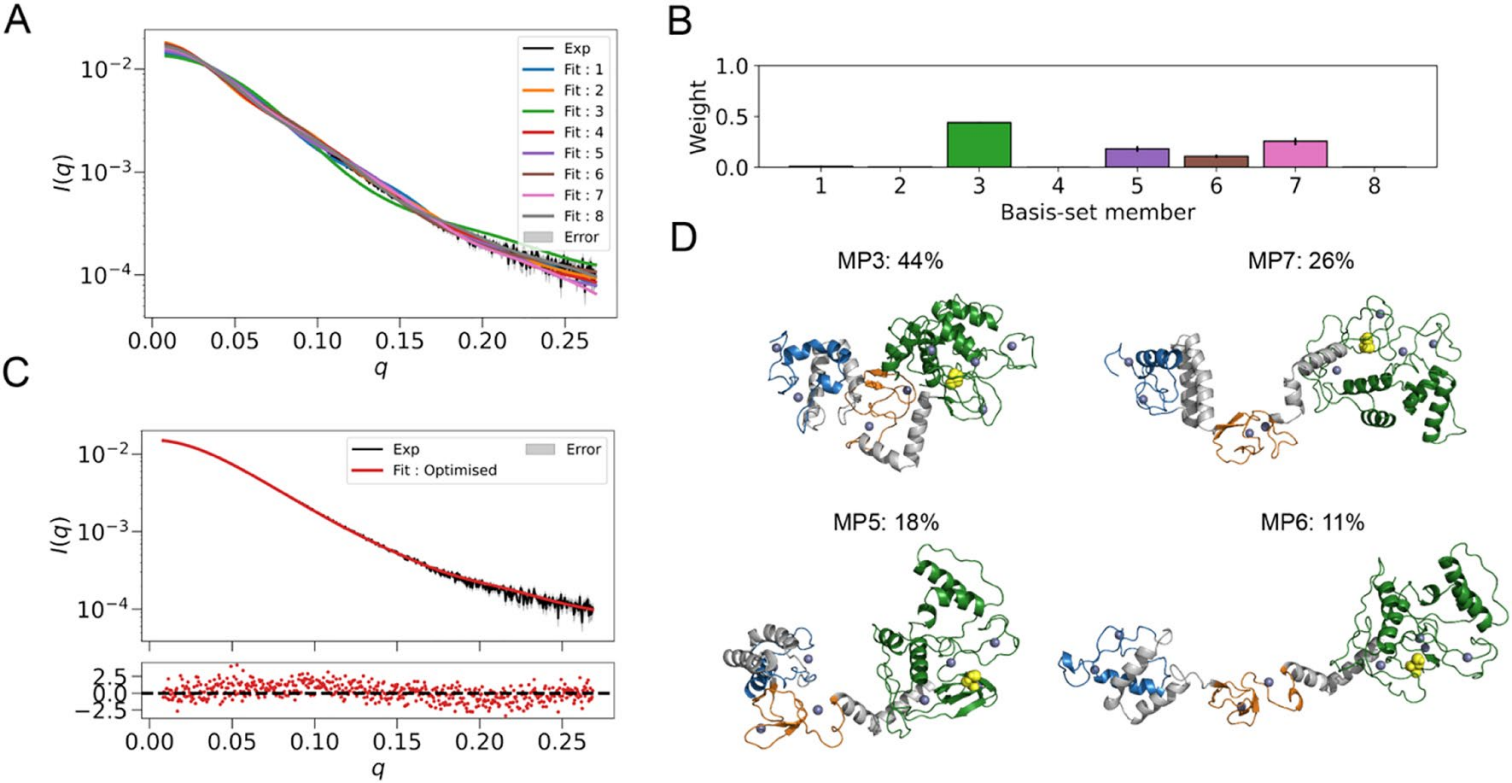
MaxPars ensemble modelling of the HOIP RBR domain. (**A**) Scattering profiles of basis-set members. (**B**) Populations of individual basis-set members, where error bars are standard deviation of the sampled posterior distribution. (**C**) Agreement between experimental and MaxPars ensemble scattering profiles. The experimental and MaxPars scattering profiles are coloured black and red, respectively. Experimental error is denoted in grey. The error-weighted residuals (I∆)/σ_exp_ = [I_exp_ (q) − I_ensemble_ (q)]/σ_exp_ versus q are plotted below. (**D**) Top 4 basis-set members with individual weights represented as percentages. RING1, IBR, RING2 and linkers are coloured in blue, orange, green and grey, respectively. The catalytic cysteine C885 is shown as yellow spheres. Zn^+2^ ions are shown as dark blue spheres. For an overlap of members in two different orientations see Supplementary Fig. 3B.

The two-step clustering approach generated a basis-set consisting of 8 members (Fig. 4A). The states MP1, MP2, MP4 and MP8 made little to no contributions between 0–1% to the final fit (Fig. 4B). The remaining 4 states MP3, MP5, MP6 and MP7 contributed most to the ensemble fit (Fig. 4B, Supplementary Figs. 3 and 4). State MP3 (*R*_*g*_ = 25.2 Å) contributed the largest weight of approximately ~ 44% to the final fit (Fig. 4B,D). States MP5 (*R*_*g*_ = 29.4 Å) and MP7 (*R*_*g*_ = 33.8 Å) made contributions of ~ 18% and ~ 26%, respectively, (Fig. 4B,D). The final state MP6 (*R*_*g*_ = 40.0 Å), the most extended conformation, made the lowest contribution to the fit of just ~ 11% (Fig. 4B,D). The optimised scattering profile of the selected basis-set indicated a good agreement with a fit of *χ*^2^_*red*_ = 1.47 to the experimental data (Fig. 4C). This suggests that the RBR domain in solution predominantly consists of more compact conformers, and that only a smaller proportion is present in extended conformations.

### MaxEnt ensemble modelling of the HOIP RBR domain

**Figure 5:**
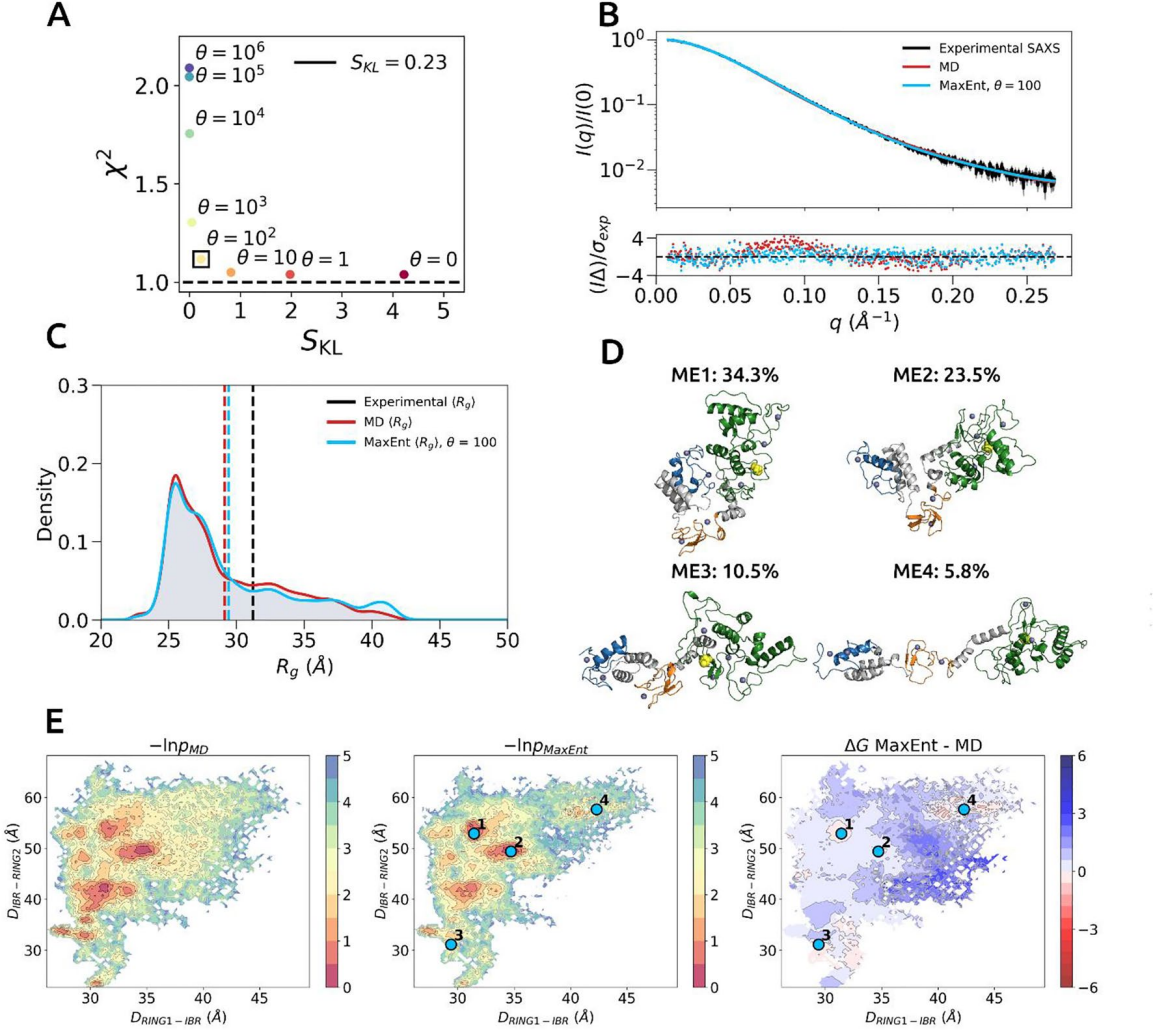
MaxEnt ensemble modelling of the HOIP RBR domain. (**A**) L-curve analysis for determining optimal value of the confidence parameter θ. The elbow of L-curve was selected as θ = 100 (black square). (**B**) Agreement between experimental and ensemble scattering profiles. The experimental, MD and MaxEnt scattering profiles are coloured black, red and cyan, respectively. The error-weighted residuals (I∆)/σ_exp_ = [I_exp_ (q) − I_ensemble_ (q)]/σ_exp_ versus q are plotted below. (**C**) Comparison between ensemble R_g_ distributions. The average values are denoted as vertical dashed lines. (**D**) Top 4 conformers (ME1-4) after clustering of structures with optimized weights (θ = 100) above one standard deviation of the mean. Percentages represent proportion of significant conformers. Structures are coloured with respect to their RING1 (blue), IBR (orange) and RING2 (green) domains. The catalytic cysteine C885 is shown as yellow spheres. Zn^+2^ ions are shown as dark blue spheres. For an overlap of members in two different orientations see Supplementary Fig. 3C. (**E**) MaxEnt reweighing of relative free energy surface. Left-hand panel represents initial MD ensemble. Middle panel represents MaxEnt reweighing of the MD ensemble. Top 4 conformers of are mapped as cyan coloured points, respectively. The right-hand panel represents difference between MaxEnt and initial MD ensembles.

Previous work suggested that the minimal ensemble approach might not be an optimal choice for systems exhibiting high conformational flexibility^26^. Thus, we decided to also construct an ensemble including all MD simulated frames. To achieve this, we used a MaxEnt reweighing framework as implemented in the BioEn software package that allows simultaneous fitting of a large number of conformers to experimental data^36^. Optimal distribution is achieved by adding minimal perturbations under constraints, where perturbations are measured in relative entropy needed to bias the initial distribution. Using BioEn, we initialized uniformly distributed weights for respective scattering curves of all MD simulated conformers. Next, the weights were refined by optimizing the negative log posterior under different values of the *θ* confidence parameter (Fig. 5A). To identify a suitable confidence *θ* parameter, we performed an L-curve analysis^36,37^, in which relative entropy values *S*_*KL*_ versus *χ*^2^_*red*_ as a function of *θ* parameter are plotted. At larger values of *θ,* optimisation does not lead to an improvement of a fit. In contrast, decreasing the value of *θ,* considerably improves the *χ*^2^_*red*_ value, however at the expense of a relative entropy increase. The optimal solution lies within the elbow region, where intermediate *θ* values simultaneously lead to a better agreement with the experimental data and little increase in relative entropy. The elbow region corresponding to a *θ* value of 100 provided a good compromise between reducing *χ*^2^_*red*_ without biasing the initial ensemble weights too much as indicated by the increase in the relative entropy (Fig. 5A). The ensemble generated using *θ* = 100 value weights was in excellent agreement with experimental data at *χ*^2^_*red*_ = 1.12 (Fig. 5B). Inspection of ensemble *R*_*g*_ distributions indicated that the RBR domain mainly populated compact conformational states that were described by *R*_*g*_ values between 25–30 Å (Fig. 5C). Nevertheless, the distribution tail contained a fraction of states with *R*_*g*_ values above 30 Å and up to around 45 Å.

There was no considerable difference between MD and MaxEnt ensembles in terms of *R*_*g*_ distributions, which indicates the appropriateness of the initial sampled ensemble. For visualising MaxEnt structural clusters, all conformers with weights above one standard deviation from the mean were termed significant^38^. Extraction of significant clusters allowed investigation of conformations that were significantly up-weighed during ensemble refinement. The significant conformers (4853 conformers) were clustered and the top four representative cluster members were extracted (Fig. 5D, Supplementary Figs. 3 and 4) (see *Trajectory Clustering* in “Methods” section). Two clusters, ME1 (*R*_*g*_ = 25.5 Å) and ME2 (*R*_*g*_ = 26.9 Å) had an abundance 34 and 24%, respectively, while the other two clusters, ME3 (*R*_*g*_ = 29.1 Å) and ME4 (*R*_*g*_ = 40.1 Å) contributed only ∼10% and ∼6%, respectively (Fig. 5D). To identify local free energy changes, a *difference* landscape was calculated by subtracting initial from reweighed landscape, where up-weighed and down-weighed regions were coloured in red and blue, respectively (Fig. 5E, middle and right panels). When inspecting free energy surfaces, it became apparent that there was a preference for compact conformational states represented by cluster ME1 and cluster ME2 (Fig. 5E, right panel). In addition, a small region of extended conformations (*D*_*RING*1−*IBR*_* ≈* 43 Å and *D*_*IBR*−*RING*2_ *≈* 58 Å) represented by cluster ME4 was up-weighed in the optimised ensemble (Fig. 5E, right panel).

Next, we wondered how many ensembles are required to achieve a good fit to the experimental data, and if 10 MD simulations were indeed necessary. To test this we optimized a collection of ensembles: all individual simulations (1–10) and several random combinations for 2-sized, 3-sized and 5-sized ensembles (Supplementary Table 3 and 4, Supplementary Figs. 5–8). The scattering profiles of individual ensembles indicated highly variable (good to poor) fitting to the experimental data with reduced χ2 values ranging from ~ 1.3 to ~ 6.3 (Supplementary Table 3, Supplementary Fig. 7). Instead, to achieve a reliably good fit with experimental data, it was necessary to include multiple simulations in the ensemble (Supplementary Table 3 and Supplementary Fig. 6). Our calculations show that at least 3, but better 5, simulations were needed to achieve good to excellent agreement with the experimental data, showing reduced χ2 values of ~ 1.1–1.2 (Supplementary Table 3 and 4, Supplementary Figs. 5 and 6).

### Comparison of MaxPars and MaxEnt ensembles

**Figure 6:**
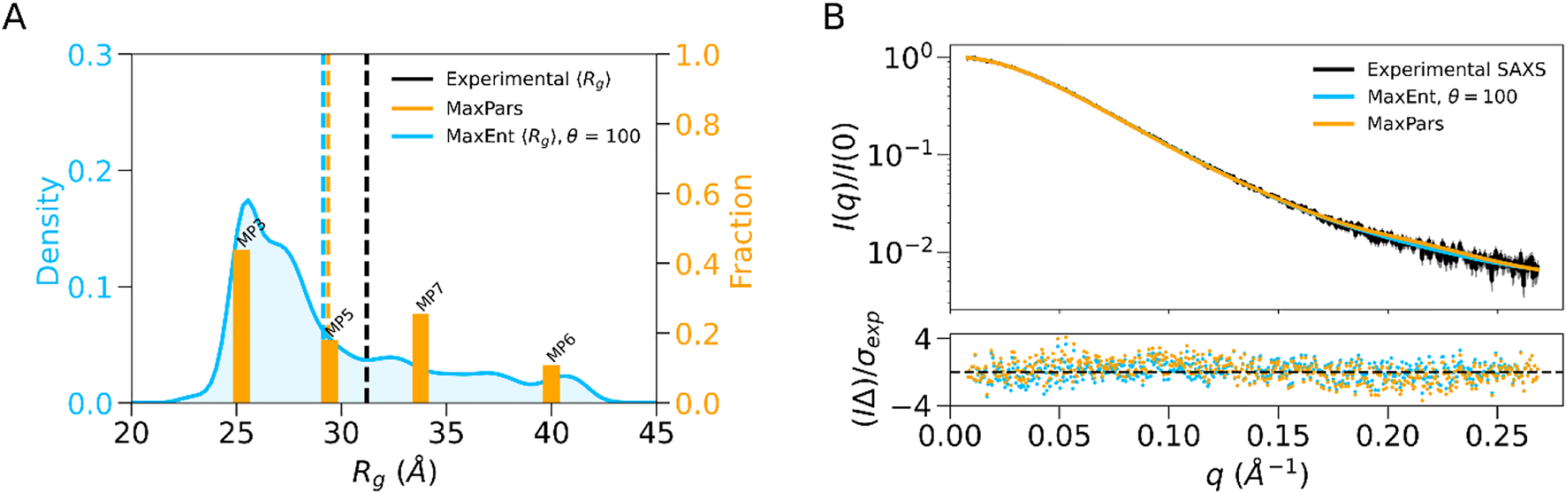
Comparison of MaxPars and MaxEnt ensembles of the HOIP RBR domain. (**A**) MaxPars and MaxEnt are visualised as discrete and continuous *R*_*g*_ distributions, respectively. The average values are denoted as vertical dashed lines. (**B**) Agreement between experimental and ensemble scattering profiles. The experimental, MaxEnt and MaxPars scattering profiles are coloured black, light blue and orange, respectively. The error-weighted residuals (I∆)/σ_exp_ = [I_exp_ (q) − I_ensemble_ (q)]/σ_exp_ versus q are plotted below.

A comparison of MaxPars and MaxEnt ensembles showed that both approaches reach the same general conclusions with the relative conformers exploring closely releated minima on the energetic landscape (Supplementary Fig. 4). To compare ensembles, we plotted R_g_ values of the top 4 representative cluster members with their associated MaxPars-inferred weights as a discrete distribution alongside the continuous distribution of the MaxEnt ensemble (Fig. 6A). There was a predominance of compact and semi-compact conformers characterised by R_g_ values between 25–30 Å in MaxEnt ensemble (Fig. 6A). The 25–30 Å region of the MaxEnt ensemble R_g_ distribution coincided with the MP3 and MP5 states, which make the largest contribution (~ 65%) to the fit of the MaxPars ensemble (Fig. 6A). Similarly, MP6 and ME4 states representing extended conformations, contribute only a small percentage to the overall fit (Figs. 4D,5D,6A). Lastly, we compared ensemble R_g_ averages with the experimentally determined value. The computed R_g_ averages of both MaxPars and MaxEnt ensembles were 29.4 Å, which were slightly closer to the SAXS-based *P(r)*-derived R_g_ value of 31.2 +/− 0.1 Å than the MD ensemble average (29.1 +/− 1.1 Å), thus indicating an improved fit (Fig. 6B).

## Discussion

RBR ligases transfer ubiquitin in a multi-step reaction that requires significant rearrangements of the three subdomains with respect to one another and is enabled by the flexible nature of the linkers connecting the subdomains. For HOIP, only two crystal structures of the entire RBR domain are available, the active form bound to an E2 ~ Ub conjugate, and in complex with a dAb (domain antibody) ^18,21^. The E2 ~ Ub bound structure shows two different arrangements of the RBR domain which we refer to as the extended and closed forms, while the dAb bound complex also adopts an elongated RBR arrangement, which however is very different from the E2-bound arrangement due to linker twisting induced by dAb binding. Crucially, the structures of individual subdomains are maintained in these different conformations, and the changes observed occur in the linker regions. In contrast, a solution-based SAXS analysis of the HOIP RBR domain, on its own and in complex with an E2 conjugate, indicated that the RBR domain remains flexible and populates mainly elongated conformations, even when bound to an E2 ~ Ub conjugate. To interrogate these differences, we employed integrative modelling which combines experimental and theoretical data to produce consistent models of dynamic biomolecular structures. Here, we have applied a posteriori ensemble reweighing to integrate MD simulations and solution scattering data to reconcile the apparently conflicting data from crystal structures and solution-based experiments. In a posteriori reweighing, the statistical weights of ensemble conformers generated from unrestrained simulations are modified to obtain a better matching ensemble average to the experimental data^25,26,39,40^. The usefulness of this approach is its simplicity in specifying a forward model for computing experimental observables and low computational costs associated with ensemble refinement. Specifically, we applied two distinct a posteriori ensemble reweighing frameworks: maximum parsimony (MaxPars) and maximum entropy (MaxEnt). MaxPars reweighing follows the principle of Occam’s razor. The optimal MaxPars solution is the ensemble containing the minimum number of structures needed to explain experimental data^26^. In MaxEnt, the optimal distribution is achieved by minimally perturbing the initial distribution while imposing certain constraints, where the magnitude of perturbation is measured by the amount of relative entropy needed to bias the distribution^36,41,42^⁠. To avoid biasing ourselves towards a particular method, we compared the ability of both approaches to describe the conformational dynamics of the HOIP RBR domain and interpret experimental SAXS-derived data.

The initial MD simulation analysis of the HOIP RBR domain highlighted that most of the ensemble is populated with compact conformers with shorter interdomain distances that coexist with a smaller proportion of extended conformations (Fig. 3B). Even after MaxPars reweighting, the ensemble consists of largely compact and semi-compact structures (Fig. 4D). Equally, the MaxEnt ensemble points to similar conformational preferences where only a small proportion of the HOIP RBR domain adopts extended structures (Fig. 6). Despite the differences between MaxPars and MaxEnt ensemble solutions, the two approaches reach similar conclusions, indicating that conformational preferences of the HOIP RBR domain are inferred from the fitting to the experimental data rather than from any inherent assumptions of the integrative modelling frameworks. Out of the two reweighing methods, we found that the MaxEnt ensemble produced an overall better fit. This observation agrees with previous studies suggesting that systems exhibiting a high degree of conformational heterogeneity, i.e., high entropy, should be studied using MaxEnt-based approaches^26^. Further investigation of this method, highlighted that multiple MD simulations are needed to obtain reliably good fits but that combination sets of 5 simulations are already, in this case, sufficient to obtain excellent agreement with the experimental data.

In summary, we have combined SEC-SAXS experiments and MD simulations to investigate the conformational behaviour of the catalytic HOIP RBR domain in solution. This study shows that the multi-domain RBR domain exists in a conformational equilibrium that is dominated by conformational states with shorter interdomain distances between RING1 and RING2, however with highly varied relative orientations of the subdomains to each other due to twisting of the linker regions. More elongated conformations make only a smaller contribution to the overall ensemble. This observation indicates that our previous SAXS-based ab-initio modelling^23^ which concluded that the RBR domain primarily populates elongated conformational states in solution overestimated their proportion within the overall conformation ensemble and emphasizes the power of integrated modelling approaches to investigate multi-domain protein conformational dynamics and help interpret experimental data^28,43^. Furthermore, our MD simulations highlight the inherent flexibility of RBR interdomain linkers that allow the subdomains to twist and rearrange and adopt multiple different conformational states. Such flexibility is likely important to enable RBR ligases to respond to a multitude of cellular regulatory signals including association with E2 ~ Ub conjugates, phosphorylation or allosteric activation by ubiquitin and possibly other molecules and modulate catalytic activity according to the specific input received.

## Methods

### Protein production and purification

The HOIP-RBR (aa 697–1072) construct used in this study was described previously^44^. Protein was expressed in LB medium supplemented with 200 µM ZnCl_2_ in BL21(Gold)DE3 *Escherichia coli* cells and induced with 0.5 mM IPTG at 18 °C for 14–16 h. Protein purification followed a 3-step purification protocol consisting of an affinity step using Glutathione Sepharose 4 Fast Flow, followed by GST-tag removal with with 3C-protease and ion-exchange chromatography using a HiTrap Q Fast Flow column. The protein was further purified by size-exclusion chromatography (SEC) using a Superdex 200 column in 25 mM HEPES, 150 mM NaCl, 0.5 mM TCEP, pH 7.5. Protein concentrations were determined by UV spectroscopy at 280 nm using ExPASy ProtParam calculated extinction coefficients (https://web.expasy.org/protparam/). Concentrated proteins were flash frozen in liquid nitrogen. Protein molecular mass was verified by electrospray ionization mass spectrometry.

### Size-exclusion small angle X-ray scattering (SEC-SAXS)

SEC-SAXS data were collected at SOLEIL Light Source on beamline SWING (Supplementary Table 1). In-line SEC-SAXS was performed using an Agilent 1200 HPLC system equipped with 3 ml Agilent Bio SEC-3, 300 Å, 4.6 × 300 mm, 3 µm, HPLC column. Samples were loaded onto size-exclusion column previously equilibrated with 25 mM HEPES, 150 mM NaCl, 0.5 mM TCEP and pH 7.5. Frames were collected for the duration fractionation run, starting at 7 min. Frames collected before the void volume were averaged and subtracted from the signal of the elution profile to account for background scattering. The primary data reduction of collected frames was performed using SWING beamline software Foxtrot (3.5.2-3645v) (https://www.synchrotron-soleil.fr/en/beamlines/swing). Data frames for averaging to produce final scattering profiles were selected using CORMAP⁠. Data processing was carried out with ATSAS software suite (2.8.4-1v)^45^ ⁠ to obtain the radius of gyration (*R*_*g*_), the maximum particle dimension (*D*_*max*_), the Porod volume (*V*_*p*_) and the pair distribution function (*P*_*r*_).

### Simulated systems

The extended HOIP RBR (699–1071) conformation (Fig. 2C) was prepared as a starting model for MD simulations by extracting coordinates from the X-ray structure of the HOIP RBR/E2 ~ Ub complex (Protein Data Bank entry: 5EDV)^18^ ⁠ (Fig. 2). However, both HOIP RBR chains (A and B) within 5EDV contained missing loop regions within RING1 and RING2 domains. To model the missing RING2 domain regions, 5EDV chain B region of 699–867 (RING1-IBR-L2) was overlapped with the 4LJP chain A region of 868–1071 (RING2), which had a fully resolved structure^46^⁠. In addition, a missing loop region of 751–759 (DLTDDTQLL) in the RING1 domain of 5EDV chain B was replaced by an identical region of the 5EDV chain A. The closed HOIP RBR (699–1071) model was prepared using the following steps: (i) RING1-IBR-L2 (699–852) and L2-RING2 (853–1071) substructures from the previously extended HOIP RBR domain model were extracted and superimposed onto the 5EDV chain B region of RING1-IBR-L2 (699–852) and 5EDV chain A region of L2-RING2 (853–936), respectively; (ii) Loop between 852–853 was closed and refined using RosettaCommons modelling suite^47^⁠ (2017.26.59567 source bundle) with Generalized Kinematic Closure and *remodel* protocols, respectively^48,49^⁠.

### All-atom simulations

The extended HOIP RBR domain (699–1071) was simulated using GROMOS 54A8 forcefield^30–32^⁠ using GROMACS 5.1.3^50^⁠ under periodic boundary conditions in a rectangular box. The solute molecules were solvated with SPC water and system charge was neutralised by replacing random waters with Cl^-^ counter ions. Before running production simulations, the system was subjected to two-stage energy minimization, followed by two-stage equilibration. The steepest-descent algorithm was applied during a two-stage energy minimization, where the system was minimized for 2,000 steps, while restraining heavy solute atoms to their initial positions using a harmonic potential with a force constant of 1,000 kJ/mol/n^2^. Followed by unrestrained energy minimization for 30,000 steps. During a two-stage equilibration procedure, the system was equilibrated for 100 ps at 50, 100, 200 and 300 K in the NVT ensemble using 10,000, 5,000, 4,000 and 1,000 kJ/mol/nm^2^ harmonic restraints on atom positions, respectively. This was followed by additional equilibration for 200 ps at 50, 100, 200 and 300 K in the NPT ensemble with the same harmonic restraints on heavy atom positions. Following equilibration, production run was initialized with generated velocities according to a Maxwell distribution at temperature of 300 K and simulated under NPT ensemble. Temperature was maintained at 300 K using velocity rescaling with a stochastic term (modified Berendsen thermostat) algorithm^51^ at coupling constant *τ*_*T*_ of 0.1 ps. Pressure was maintained isotropically at 1 bar using Parrinello-Rahman algorithm^52,53^ at coupling constant *τ*_*P*_ of 2 ps. Equations of motion were integrated using leap-frog algorithm with a 2 fs time step and neighbour search was performed using Verlet algorithm. The van der Waals interactions were cut-off at 0.9 nm. Electrostatics were calculated with the particle mesh Ewald method with a 0.9 nm cut-off. All covalent bonds were constrained using LINCS algorithm. The geometry of the simple point charge water molecules was constrained using SETTLE. Removal of center-of-mass translational and rotational velocity was done every 10 fs. Zn^2+^ ions were restrained to their respective Zn^2+^-coordinating residue pairs using distance restraints. The system was simulated 10 times for 100 ns, resulting in a cumulative simulation time of 1 µs. For all trajectories, the coordinates for analysis were saved every 20 ps, resulting in 50,001 frames.

### Trajectory clustering

Clustering of trajectory frames was performed using *hdbscan* (0.8.22v)^54,55^ with the parameter *min_size_cluster* set to a value of 5, while the rest of input parameters were set to their respective default values. To capture information about relative domain orientations and avoid bias in roto-translational alignment of trajectory frames, all pairwise distances between C-α atoms of RING1 (699–751), IBR (796–841) and RING2 (869–934) domains were computed and provided as input for *hdbscan* clustering. After cluster assignment, frames with assigned ids were extracted and noise (frames with cluster id of -1) was discarded. Representative conformer, or a centroid of a cluster, was computed based on the pairwise root-mean squared deviations (RMSD) between members of the cluster using the previously defined pairwise C-α distances of RBR domains. The pairwise RMSD matrix was transformed into a set of scores as a sum of matrix values along a single cluster member:

$${\sum }_{i=1}^{N}{R}_{ij}^{cluster}$$

where *R* is the pairwise RMSD matrix, *i* and *j* represent matrix member indices and *N* is the size of the cluster. The member with the lowest RMSD score is selected as the representative conformer of the cluster.

### Calculating SAXS curves

CRYSOL (2.8.4v)^56^ ⁠was used to calculate scattering intensities *I(q)* as a function of momentum transfer *q* defined as $q=4\pi sin\left(\theta \right)/\lambda$, where λ corresponds to the wavelength and 2θ is the scattering angle. The calculated scattering profiles were fitted against the provided experimental data with constant subtraction, which accounts for possible systematic errors due to mismatched buffers in the experimental data. Given the automatic scattering fit to the experimental data, CRYSOL calculations required input of four parameters, namely maximum order of harmonics, order of Fibonacci grid, solvent density, and contrast of hydration shell. The contrast of hydration shell was set automatically by CRYSOL due to an automated fit to the experimental data. The solvent density was set to a default value of 0.334 e/Å^*3*^*.* The order of Fibonacci grid was set to a default value of 17. The maximum order of harmonics value was set to 25. The quality of the fit was calculated using the following metric:

$${\chi }^{2}\left(q\right)={\sum_{{q}_{min}}^{{q}_{max}}\left(\frac{{I}_{\mathrm{exp}}\left(q\right)- {I}_{comp}\left(q\right)}{{\sigma }_{exp}}\right)}^{2}$$

where *I*_*comp*_*(q)* is the computed scattering intensities, *I*_*exp*_*(q)* is the experimental scattering intensities and *σ*_*exp*_ is the experimental error. The interpretation of value depends on the number of data points (*N)* used in calculation, thus a reduced ${\chi }_{red}^{2}=\left({\chi }^{2}/N\right)$ is reported, where the value close to 1 indicates a perfect fit.

### Maximum parsimony ensemble modelling

The BSS-SAXS modelling consists of three steps: structural clustering, scattering clustering and weight inference. In the first step, the structural clustering was performed as indicated in the *Trajectory Clustering* section (see “Methods” section). In the second step, for each representative cluster conformer, we compute a theoretical scattering profile and fit against the provided experimental scattering profile using CRYSOL (see *Calculating SAXS curves* in “Methods” section). The resulted scattering profiles were hierarchically clustered using Ward’s linkage based on a pairwise similarity measure:

$${\chi }_{\left(i,j\right)}^{2}\left(q\right)= \sum_{{q}_{min}}^{{q}_{max}}{\left(\frac{{I}_{i}(q) - {I}_{j}(q)}{{\sigma }_{exp}}\right)}^{2}$$

The number of clusters was defined using distance threshold of 0.15. The two-step clustering protocol yields a set K of conformationally unique and experimentally distinguishable states, which is termed basis-set. In the last step, a weighted average of theoretical scattering profiles within the basis-set is computed:

$${I}_{comp}({\varvec{w}}, q)={\sum }_{k=1}^{N}{w}_{k}{I}_{k}\left(q\right)$$

where ***w*** is a vector of weights, *I*_*k*_*(q)* are the scattering intensities of a state K and *w*_*k*_ is an associated weight. To determine relative populations of *w*_*k*_, Bayesian Monte Carlo is used to map a posterior probability of weights defined using Bayes’ Theorem:

$$P({\varvec{w}}|data) \propto P(data|{\varvec{w}})P({\varvec{w}})$$

where *P(w)* is a prior uniform distribution of weights and *P(data|w)* is a likelihood function. To account for uncorrelated and normally distributed errors, the likelihood function is defined as:

$$P\left(data|{\varvec{w}}\right)={\varvec{e}}{\varvec{x}}{\varvec{p}}\left(-{\chi }^{2}\left({\varvec{w}}\right)\right)$$

where χ^2^ is defined as:

$${\chi }^{2}\left({\varvec{w}}\right)={\sum_{{q}_{min}}^{{q}_{max}}\left(\frac{{I}_{\mathrm{exp}}\left(q\right)- c{I}_{comp}\left({\varvec{w}}, q\right)}{{\sigma }_{exp}}\right)}^{2}$$

where *c* is a scaling constant defined as^57^:

$$c = \left[\sum_{{q}_{min}}^{{q}_{max}}\frac{{I}_{exp}{I}_{comp}}{{\sigma }_{exp}^{2}}\right]{\left[\sum_{{q}_{min}}^{{q}_{max}}\frac{{I}_{comp}{I}_{comp}}{{\sigma }_{exp}^{2}}\right]}^{-1}$$

To map the posterior distribution, we conducted a Metropolis sampling for 51,000 steps, where the initial 1,000 steps were discarded as a burn-in period. We report fractional weights w_k_ and associated uncertainties as the mean and standard deviation of the observed posterior distribution, respectively.

### Maximum entropy ensemble modelling

We used Bayesian Inference of Ensembles (BioEn) (https://github.com/bio-phys/BioEN) package to determine statistical ensemble weights in accordance with MaxEnt principle^36^. Using CRYSOL, we computed scattering profiles for each sampled MD conformation (see *Calculating SAXS curves* in “Methods” section) and optimized associated weights to match the experimental data. Within the BioEn, the ensemble distribution was described as a posterior distribution as a function of weights *w*_*k*_, where *k* is the index of ensemble members (*N* = *1, …, k*), given experimental SAXS data,

$$P\left({\varvec{w}}|data\right)\propto P\left(data|{\varvec{w}}\right)P\left({\varvec{w}}\right)$$

The ***w*** is a vector of weights, *P(w)* is a prior and *P(data|w)* is a likelihood function. Prior is given by $P({\varvec{w}}) \propto exp(-\theta {S}_{KL})$, where *S*_*KL*_ is Kullback–Leibler divergence:

$${S}_{KL} = \sum_{k = 1}^{N}{w}_{k}ln\frac{{w}_{k}}{{w}_{k}^{0}}$$

and optimized ${w}_{k}$ and reference ${w}_{k}^{0}$ weights are non-negative and normalized $\sum_{i}^{N}{w}_{i}=1$. The parameter *θ* defined our confidence in the reference ensemble. The larger *θ* values, the closer refined ${w}_{k}$ weights would be to reference ${w}_{k}^{0}$ weights. Likelihood function is implemented as $P(data|{\varvec{w}})\boldsymbol{ }\propto \boldsymbol{ }exp \left({\chi }^{2}/2\right)$. The BioEn *χ*^2^ formulation differs by a factor of ½, which is equivalent to rescaling *θ* value. Instead of maximising a posterior distribution, BioEn performs an optimisation of a negative log-posterior given by:

$$L = \theta \sum_{k = 1}^{N}{w}_{k}ln\frac{{w}_{k}}{{w}_{k}^{0}} + {\sum_{{q}_{min}}^{{q}_{max}}\left(\frac{{\sum }_{k=1}^{N}{w}_{k}{I}_{k}\left(q\right) - {I}_{\mathrm{exp}}\left(q\right)}{2{\sigma }_{exp}}\right)}^{2}$$

The multiple minimisations were performed over a set of *θ* values using a GSL optimiser. To account for nuisance parameters, such as unknown scaling parameter, BioEn performs an iterative weight optimisation, which was set to 10 iterations. To select an optimum *θ* value, the L-curve analysis was performed for *S*_*KL*_ vs. *χ*^2^ values. Once the optimum *θ* value was selected, the corresponding optimised weights ${w}_{k}$ were extracted and used to perform a structural interpretation of molecular simulations.

### Data analysis

Data analysis was performed with *python* (3.7v). Plots were generated with *matplotlib* (3.1.0v). Protein trajectories were analysed using *MDAnalysis* (0.19.2v) and *mdtraj* (1.9.3v). Error estimation was determined using block analysis ^43,58^. Protein structures were visualized with PyMOL (1.7.2v).

## Supplementary Information

Supplementary Information.

## Data Availability

The datasets generated and analysed during the current study are available in the Github repository: https://github.com/Fraternalilab/HOIP-ensembles-Kausas-et-al.
