# Supplementary material for: Characterisation of HOIP RBR E3 ligase conformational dynamics using integrative modelling
Source: Sci Rep. 2022 Sep 8;12:15201. doi: 10.1038/s41598-022-18890-6 (PMC9458678; doi:10.1038/s41598-022-18890-6)
Supplement: Supplementary file 1 — Supplementary Information. [file 41598_2022_18890_MOESM1_ESM.pdf]

Supplementary Information

Characterisation of HOIP RBR E3 ligase conformational dynamics using integrative modelling

Marius Kausas[1,2], Diego Esposito[2], Katrin Rittinger[2]* and Franca Fraternali[1,3]*

[1]New Hunt's House (room 3.14), King's College London, Guy's Campus, SE1 1UL London UK

[2]Molecular Structure of Cell Signalling Laboratory, The Francis Crick Institute, 1 Midland Road, London, NW1 1AT, United Kingdom

[3]The Francis Crick Institute, 1 Midland Road, London, NW1 1AT, United Kingdom

*Correspondence:

franca.fraternali@kcl.ac.uk

katrin.rittinger@crick.ac.uk

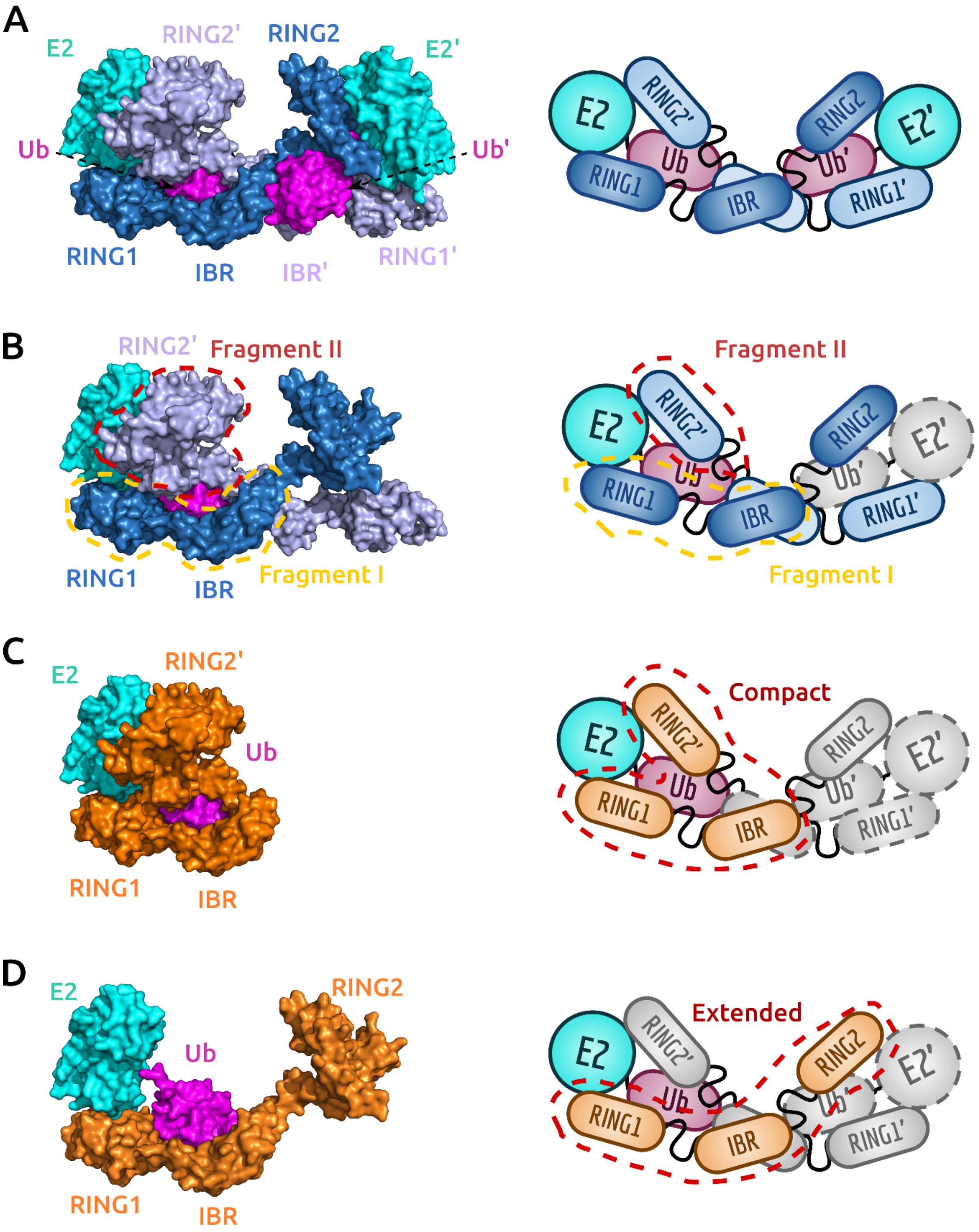

Supplementary Figure 1. Crystal structure of the HOIP RBR domain. Coordinates were extracted from Protein Data Bank entry: 5EDV (Lechtenberg et al., 2016). The visualisation of each structure is aided with a schematic drawing. (A) The asymmetric unit is composed of two HOIP RBR molecules (dark and light blue) interacting with two UbcH5B~Ub conjugates (UbcH5B is coloured in cyan; ubiquitin is coloured in magenta). (B) Contents of the

asymmetric unit without one of the UbcH5B~Ub conjugates. In the cross-dimer configuration, RING1-IBR fragment of one RBR molecule (Fragment I, outlined in yellow) together with the RING2 fragment of another RBR molecule (Fragment II, outlined in red) are interacting with one UbcH5B~Ub conjugate. (C) The *closed* conformation (orange and outlined in red) of HOIP RBR domain is composed of RING1-IBR fragment of one RBR molecule and the RING2 fragment of another RBR molecule. The other RBR regions and second UbcH5B~Ub conjugate are omitted for clarity. (D) The *extended* conformation of HOIP RBR domain (orange and outlined in red) represented by one of the RBR polypeptides seen in the asymmetric unit. The second RBR molecule and UbcH5B~Ub conjugate are omitted for clarity.

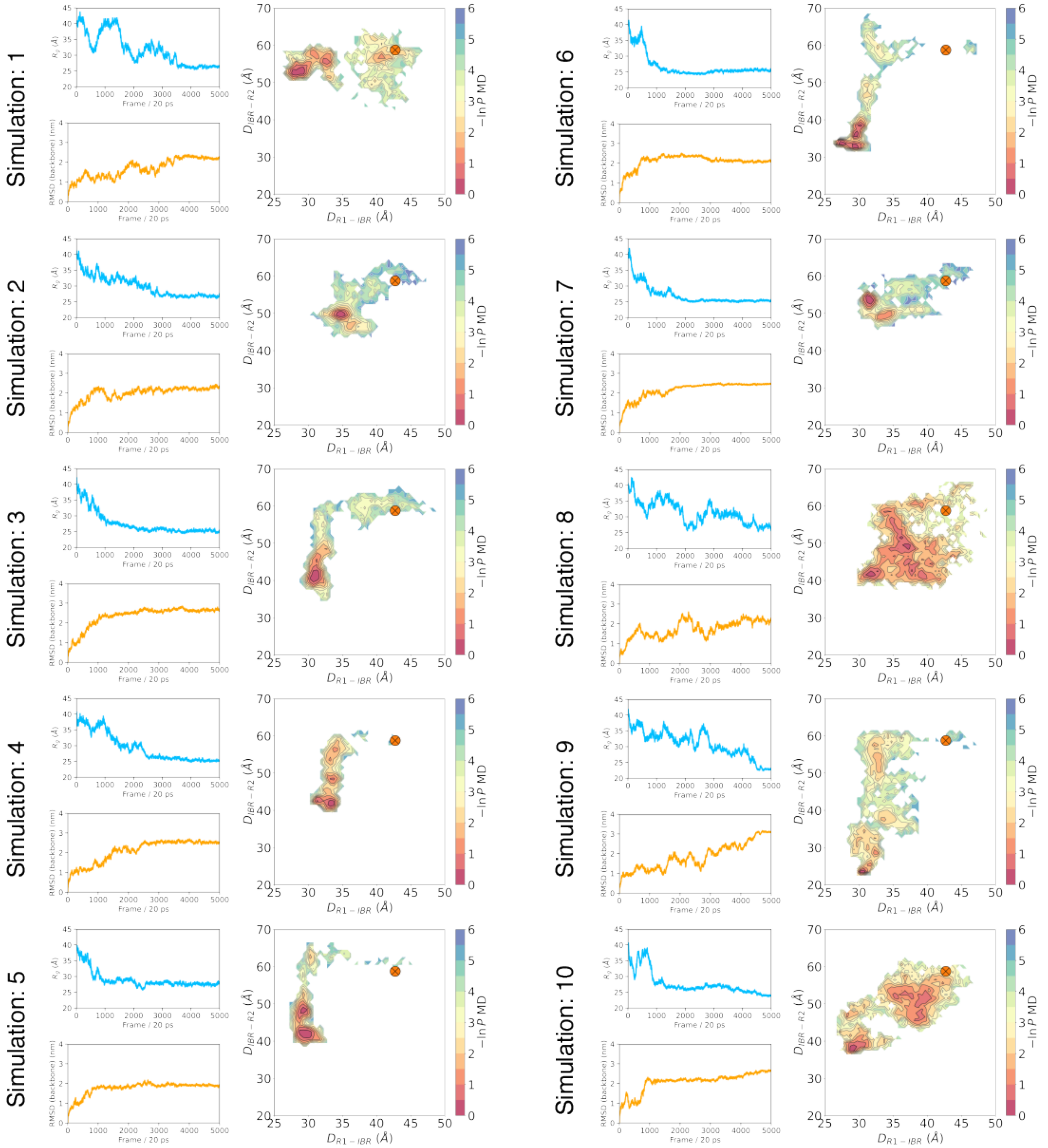

Supplementary Figure 2. Comparison of radius of gyration, root-mean-squared deviations (calculated on backbone atoms) and energy landscapes representation projected onto two collective variables, $D_{RING1\text{-}IBR}$ and $D_{IBR\text{-}RING2}$, of the extended HOIP RBR domain for each of the 10 x 100ns simulations. The starting MD conformation of the extended HOIP RBR domain is represented as an orange circle cross in the landscape representations**.**

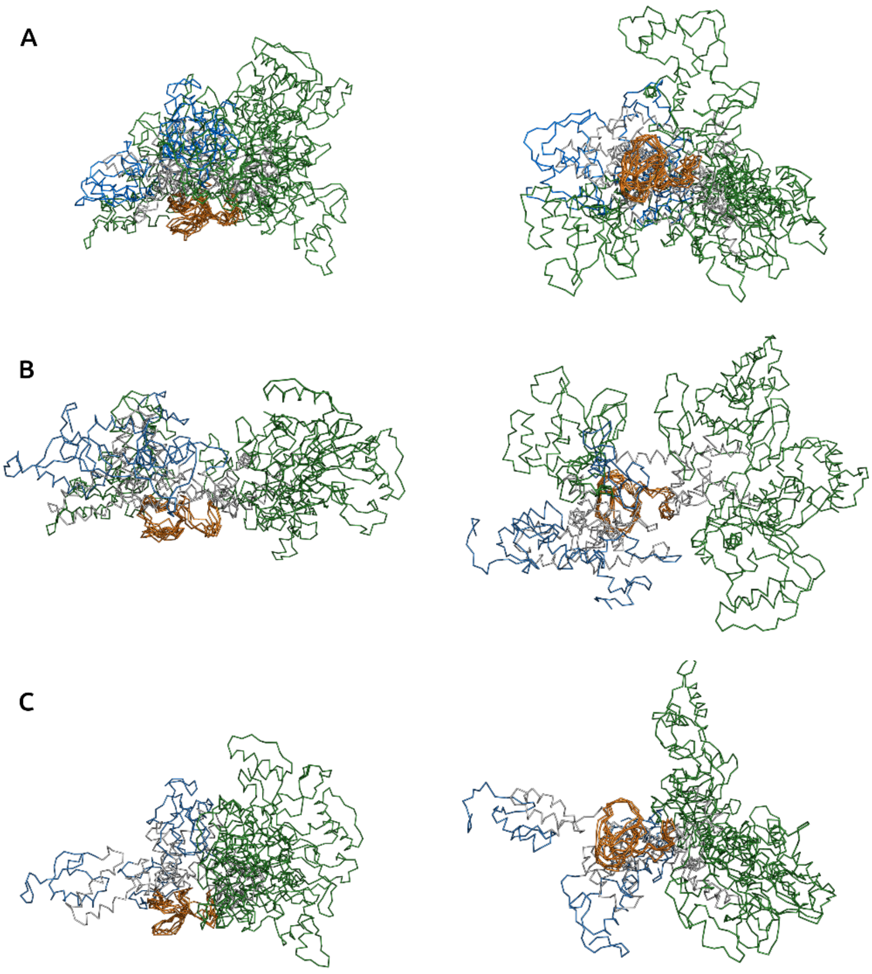

Supplementary Figure 3. Comparison of MD, MaxPars and MaxEnt ensembles. All respective ensemble conformers are aligned onto the IBR domain. The right-hand column shows the overlapped ensembles rotated by 90°. Structures are displayed as ribbons and coloured with respect to their RING1 (blue), IBR (orange) and RING2 (green) domains. (A) MD ensemble conformers (MD1-6) as shown in Figure 1C. (B) MaxPars ensemble conformers (MP3, MP5-7) as shown in Figure 4D. (C) MaxEnt ensemble conformers (ME1-4) as shown in Figure 5D.

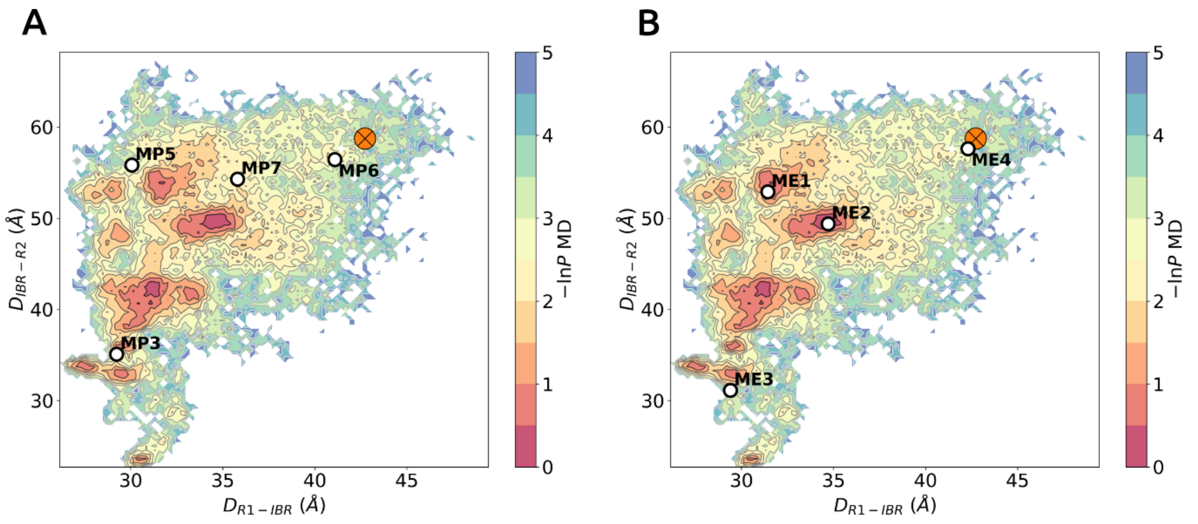

Supplementary Figure 4. Comparison of free energy surface of unweighted MD ensemble with MaxPars and MaxEnt ensemble members. (A) MaxPars ensemble conformers (MP3, MP5-7) and (B) MaxEnt ensemble conformers (ME1-4) are plotted as white points onto the projected free energy surface of collective variables, $D_{RING1-IBR}$ and $D_{IBR-RING2}$. Starting MD conformation of the extended HOIP RBR domain for molecular simulations is represented as orange circle cross.

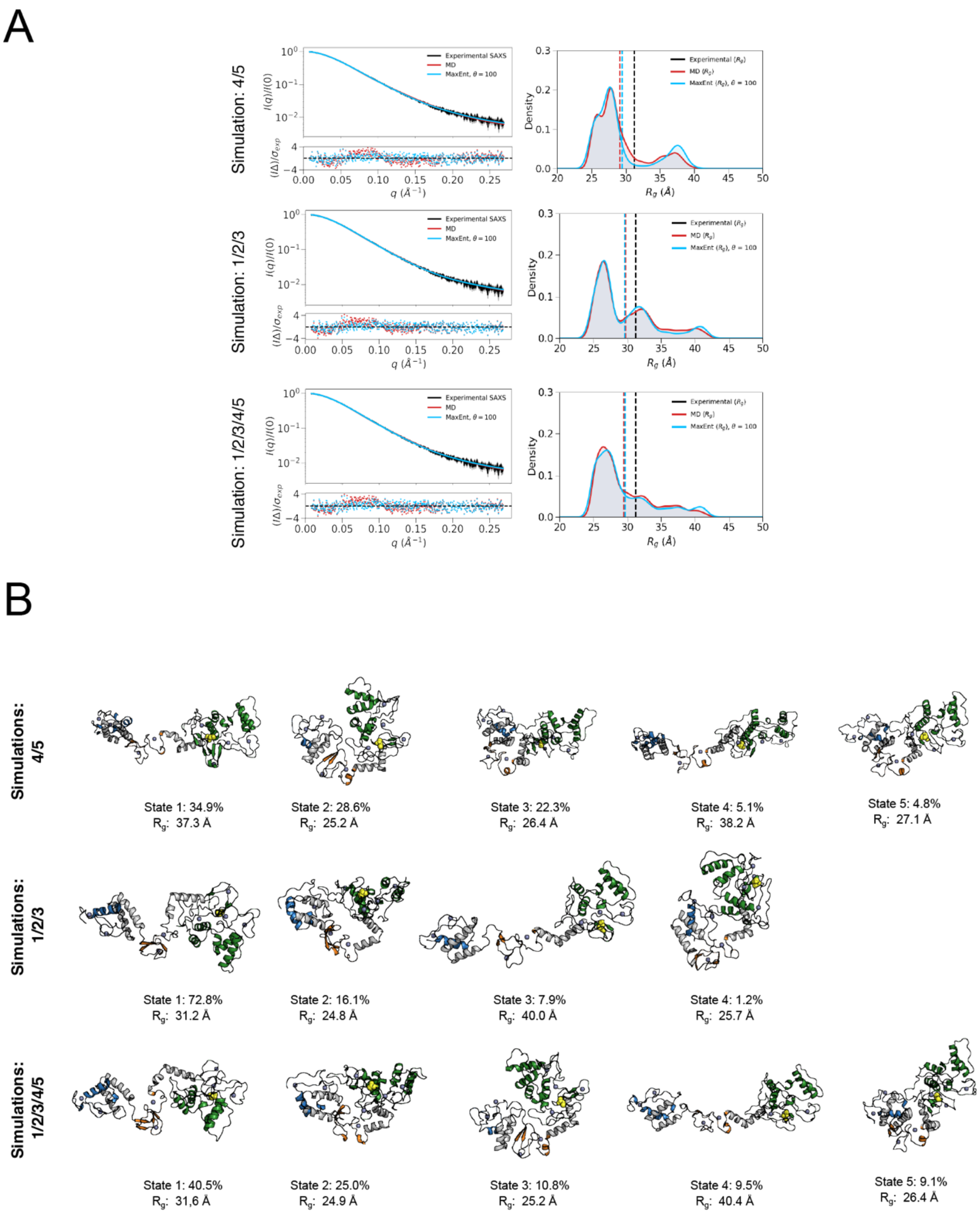

Supplementary Figure 5. (A) Comparison of maximum entropy optimized ensemble fits and radius of gyration distributions for selected simulation combinations.
(B) Significant states after structural clustering of conformers with one standard deviation above the mean of optimized weights for selected simulation combinations.

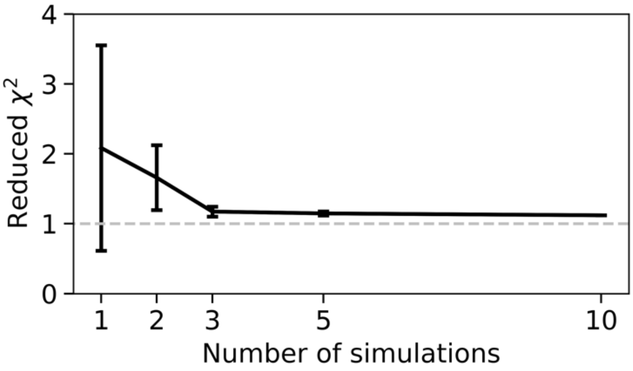

Supplementary Figure 6. Comparison of mean reduced χ^2 values of simulation combinations for 1-sized, 2-sized, 3-sized, 5-sized and full (10-sized) ensembles. Error bars represent standard deviation.

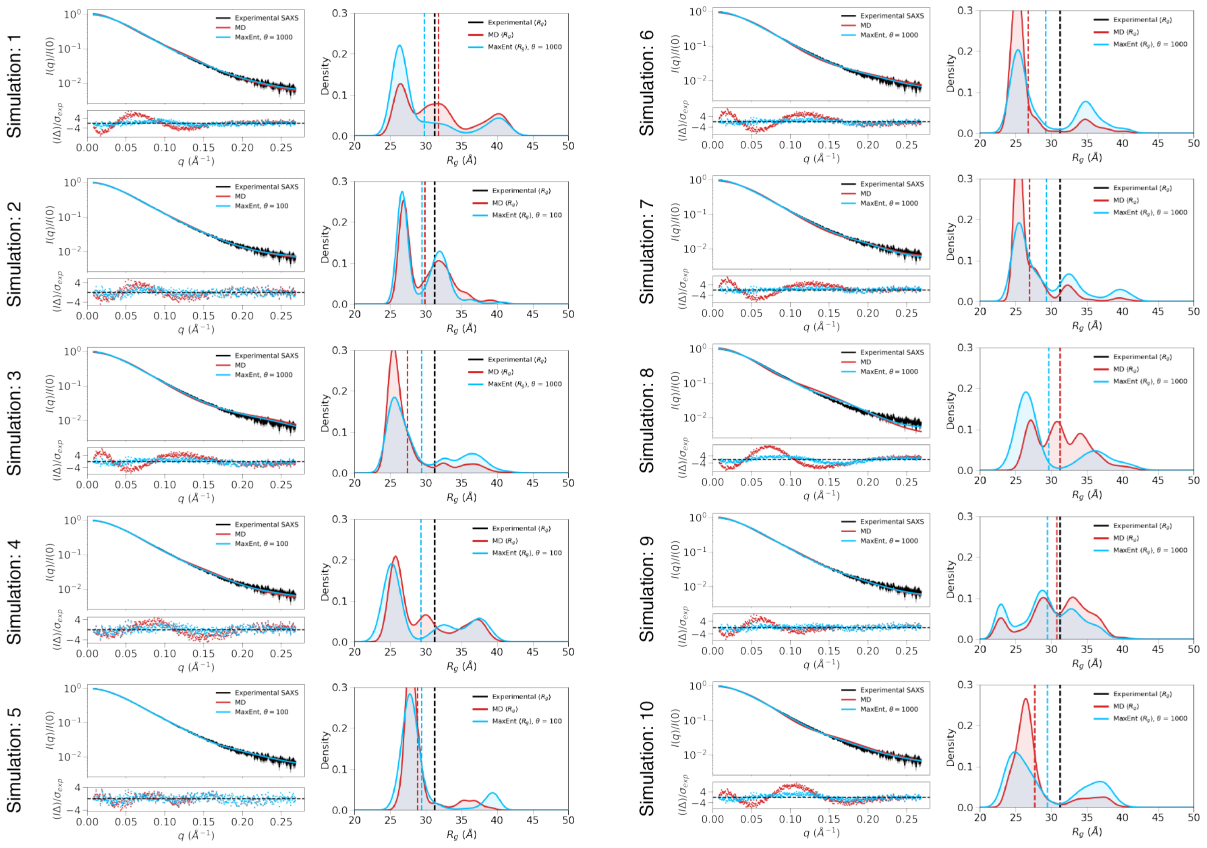

Supplementary Figure 7. Comparison of maximum entropy optimized ensemble fits and radius of gyration distributions for each individual simulation.

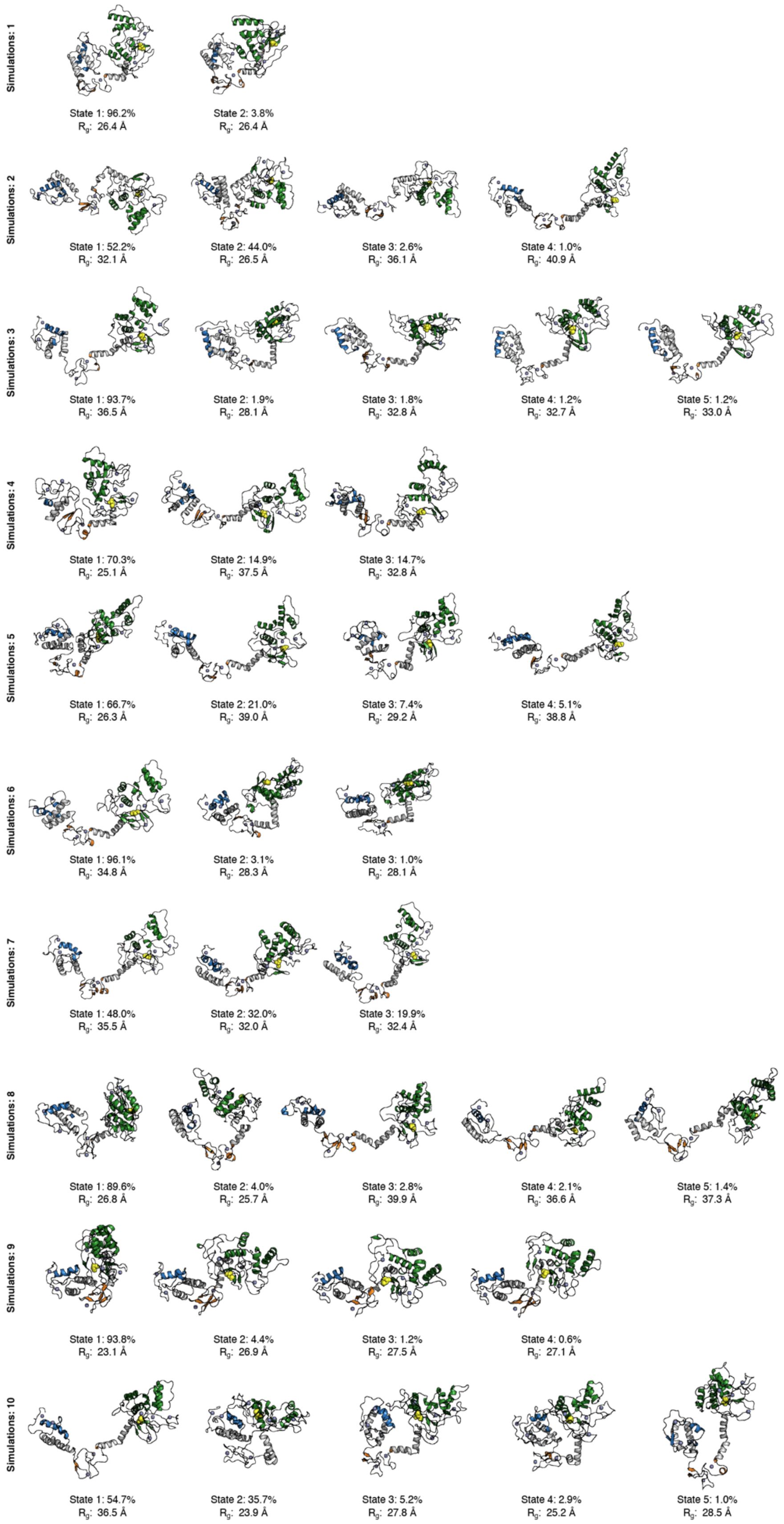

Supplementary Figure 8. Significant states after structural clustering of conformers with one standard deviation above the mean of optimized weights for each individual simulation.

**Data collection**	
Beamline	SWING Beamline, SOLEIL
Beam energy (keV)	12
Wavelength (Å)	1.03318547479
Sample-detector distance (m)	2
Detector	Eiger-4M (DECTRIS)
Pixel size (μm^2)	75 × 75
Exposure time (ms)	990
Number of exposures	images taken for the duration of the run, starting at 7 min
Sample storage temperature (K)	283.15
Column temperature (K)	288.15
q-measurement range ($Å^{-1}$)	0.004-0.54
**SEC-SAXS**	
Buffer	25 mM HEPES, 150 mM NaCl, 0.5 mM TCEP, pH 7.5
SEC column	Bio SEC-3 300˚*A*
Flow rate (ml/min)	0.3
**Data analysis**	
Primary data reduction	Foxtrot (3.5.2-3645)
Guinier, $P(r)$, Porod volume (V_p)	ATSAS (2.8.4-1)

Supplementary Table 1: SAXS data collection parameters.

**Sample parameters**	
Sample	HOIP RBR (697-1072)
Injected concentration (mg/ml)	9.0
Injection volume (μl)	70
**Guinier analysis**	
$I(0)$ (cm^{-1})	0.015 ± 2.3*e*-05
R_g (Å)	30.3 ± 0.1
q-range (Å^{-1})	0.0082-0.0356
Points used for Guinier analysis	5-66
qR_g limit	1.09
***p*(*r*) analysis**	
$I(0)$ (cm^{-1})	0.015 ± 2.2*e*-05
R_g (Å)	31.2 ± 0.1
D_{max} (Å)	120
q-range (Å^{-1})	0.0082-0.2684
Range of *q*-points	7-576
Number of points in real space	191
Alpha (GNOM)	70.89
Total estimate from GNOM	0.80
V_P Porod volume (Å^3) [a]	65300
**Molecular weight estimation (kDa)**	
MW from sequence	43.4
MW from SAXS MoW2 [b]	45.7
MW from V_P Porod volume [c]	40.8

(a) Porod volumes were calculated using GNOM in the ATSAS package.
(b) Molecular weights were estimated using SAXS MoW2 with a threshold of $Q_{max} = 8/R_g$.
(c) Molecular weights were obtained by dividing Porod volume by 1.6.

Supplementary Table 2: SAXS data analysis of the HOIP RBR domain.

Simulation Identifier	Reduced χ2	Simulation combina-tions sets	Reduced χ2	Simulation combinations sets	Reduced χ2
**1**	1.87	**1, 2**	1.59	**2, 3, 7**	1.12
**2**	1.24	**3, 6**	1.73	**1, 9, 10**	1.16
**3**	1.85	**4, 5**	1.12	**1, 2, 3, 4, 5**	1.12
**4**	1.38	**8, 10**	2.50	**6, 7, 8, 9, 10**	1.18
**5**	1.25	**7, 9**	1.16	**1, 3, 5, 7, 9**	1.12
**6**	1.62	**6.8**	1.12	**2, 4, 6, 7, 10**	1.12
**7**	1.41	**1, 2, 3**	1.13	**3, 4, 7, 8, 10**	1.19
**8**	6.34	**4, 6, 9**	1.16	**1, 3, 4, 6, 9**	1.15
**9**	1.31	**5, 6, 7**	1.33	**1, 2, 3, 4, 5, 6, 7, 8, 9, 10**	1.12
**10**	2.57	**2, 4,10**	1.14		

Supplementary Table 3. Comparison of maximum entropy optimized ensemble fits for individual simulations and combinations thereof.

Ensemble size	Mean reduced χ2 values	Standard deviation of re-duced χ2 values
1	2.08	1.47
2	1.66	0.46
3	1.17	0.072
5	1.15	0.029
10	1.12	0.0

Supplementary Table 4. Comparison of mean ensemble fit values of combinations for 1-sized, 2-sized, 3-sized, 5-sized and full (10-sized) ensembles.
